# In vivo assessment of number of milk duct orifices in lactating women and association with parameters in the mother and the infant

**DOI:** 10.1186/1471-2393-14-124

**Published:** 2014-04-02

**Authors:** Julia Jütte, Ariane Hohoff, Cristina Sauerland, Dirk Wiechmann, Thomas Stamm

**Affiliations:** 1 Private Practice, Hardter Waldstraße 24, 41169 Mönchengladbach, Germany; 2 Department of Orthodontics, University of Münster, Albert-Schweitzer-Campus 1, 48149 Münster, Germany; 3 Institute of Biostatistics and Clinical Research, University of Münster, Schmeddingstraße 56, 48149 Münster, Germany; 4 Department of Orthodontics, Medizinische Hochschule Hannover, Carl-Neuberg-Str. 1, 30625 Hannover, Germany

## Abstract

**Background:**

In vitro and in vivo analyses differ between the number of milk ducts found in the lactating breast, and there is a lack of knowledge as to whether or not external factors in the mother or the child affect the number of ductal orifices. The aim of this study was to determine the number of milk duct orifices in vivo and to investigate the possible influence of variable parameters in mother and infant.

**Methods:**

Study design: Prospective clinical trial. In 98 breastfeeding women we investigated the nipple surface in order to identify the number of milk duct orifices using Marmet’s manual milk expression technique. In addition mothers were interviewed on different parameters of birth and breastfeeding.

**Results:**

Every nipple had 3.90 ± 1.48 milk duct orifices on average. There was no significant difference between left and right breasts. The use of a breast pump in addition to breastfeeding did not have any effect on the number of ductal orifices. Multiparous women exhibited more ductal orifices (8.5 ± 3.0) as compared to primipara (7.1 ± 2.7). Boys were associated with significantly more ductal orifices in their mother’s right breast (4.2 ± 1.7) than girls (3.5 ± 1.4). Furthermore boys were breastfed for longer per session. A shorter birth height of males correlated with more ductal orifices in left nipples. Fluid intake of mothers was associated with a higher number of ductal orifices. Restless infant behavior could not be explained by less milk duct orifices. Pain in the breast during breastfeeding did not have an influence on ductal orifices either. Psychological criteria, such as duration of maternity leave and total intended breastfeeding period, did not affect the number of orifices in the papilla mammaria of breasts during lactation.

**Conclusion:**

For the first time an in vivo investigation of the number of ductal orifices in lactating women was conducted non-invasively and associations with variables in the mother and the child, birth parameters in infants, and breastfeeding parameters in mothers and children were assessed. We conclude that the number of activated ductal orifices on the surface of the nipple is primarily associated with functional aspects.

## Background

**Figure 1:**
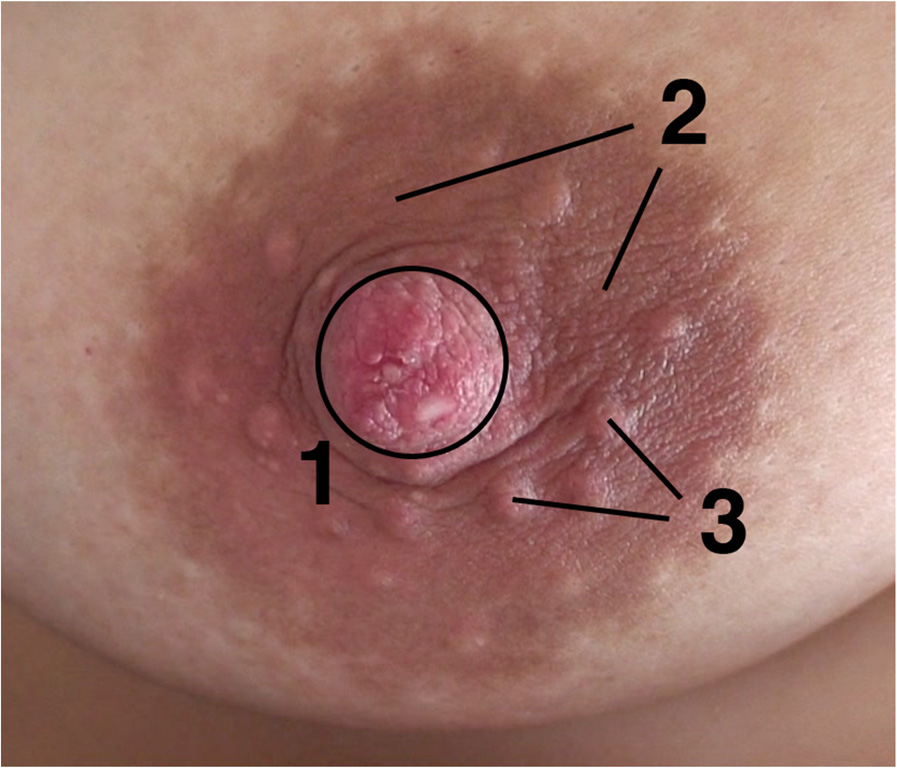
**Nipple-areola-complex.** (1) Papilla mammaria. (2) Areola mammae. (3) Tubercula areolae.

**Figure 2:**
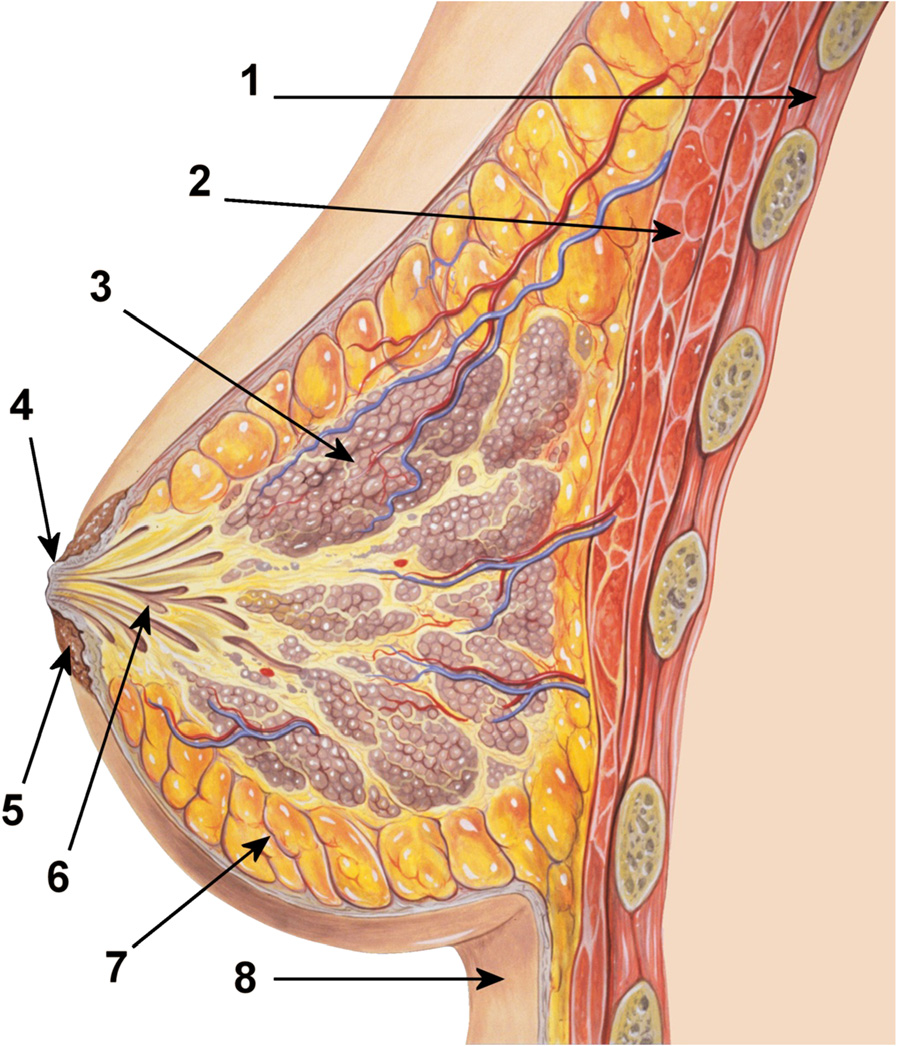
**Diagram of the ductal anatomy of the breast.** (1) Chest wall, (2) Pectoral muscles, (3) Lobules, (4) Nipple surface, (5) Areola, (6) Lactiferous duct, (7) Fatty tissue, (8) Skin (original author: Patrick J. Lynch; reworked by Morgoth666 to add numbered legend. (Patrick J. Lynch, medical illustrator) [CC-BY-3.0 (http://creativecommons.org/licenses/by/3.0)], via Wikimedia Commons).

The female human breast extends from the second to the sixth rib while the so-called nipple-areola complex is usually located between the fourth and fifth ribs. This complex consists of the nipple, i.e., the conical papilla mammaria, and the areola mammae, a likewise pigmented area surrounding the nipple. The tubercula areolae are nodular glands visible on the areola (Figure 1). Fifteen to 20 lobes form the mammary gland consisting of various cell types embedded in adipocellular and fibrous tissue [1,2]. Milk ducts of the mammary gland course to the surface of the nipple and enable the transport of breast milk during lactation (Figure 2) [3,4].

Different examination methods exist to determine the number and morphology of the milk ducts in the lactating breast. Sonography helps identify milk ducts coursing from the base of the papilla mammaria to the deep parenchyma. Concerning the number of ductal orifices on the surface of the nipple, no results have been presented using sonography [5]. Ductoscopy requires local anesthesia before inserting microendoscopes into the orifices of the milk ducts to visualize the duct system [6,7]. Galactographic as well as mammographic examination methods require the application of x-radiation and visualize the ductal system insufficiently. Neither computer tomography nor magnetic resonance imaging have yielded results useful for determining the number of milk ducts in the human breast [8]. The microscopic evaluation of histologic sections of the female breast with its nipple-areola complex is seen as the gold standard for milk duct identification [7]. However, it cannot represent the physiologic state of the lactating breast. This emphasizes the need for a non-invasive, risk-free examination method to determine the number of milk duct orifices in the nipple of a lactating breast. In our study, therefore, we used Marmet’s manual milk expression method [9].

Insights from in vivo analysis of the ductal orifices in the nipple help understand the whole breastfeeding process and adaptive interactions between mother and child. This knowledge may help with breastfeeding problems, when mothers fear their milk will be insufficient to sustain the infant. We hypothesize that the number of active ductal orifices on the surface of the nipple is associated with functional aspects.

## Methods

Subject enrolment took place at the following Departments of Obstetrics and Gynecology: (i) Universitätsklinikum Münster, (ii) Knappschaftskrankenhaus Recklinghausen, (iii) Paracelsus Klinik Marl, and (iv) St. Vincenz Krankenhaus Datteln. All clinics are located in North Rhine-Westphalia, a federal state in the western part of Germany. All departments gave permission to conduct the study.

### Inclusion criteria

Inclusion criteria were as follows: (i) Caucasian mothers with delivery in the 37^th^ week of gestation or later; (ii) delivery without complications, for neither mother nor child; (iii) good general health status of mother and child; (iv) breastfeeding mother; and (v) mother who had given informed consent.

### Exclusion criteria

Exclusion criteria were as follows: (i) delivery before the 37^th^ week of gestation; (ii) general disease of the mother or child; (iii) mother who stopped breastfeeding; (iv) mother feeding infant with nursing bottle; and (v) mother feeling pain during manual milk expression.

Mothers who agreed to participate were interviewed on age, parity, maternity leave, use of a manual or electric breast pump, problems with breastfeeding, and fluid intake. They were also interviewed on their child’s sex, age in days, birth weight, and height.

Manual milk expression was explained by a lactation consultant demonstrating the Marmet technique [9]. The aim was to evaluate the maximum available duct orifices in the most physiological and natural scenario available. Therefore, observation took place immediately before breastfeeding. When the mother felt she had enough milk to feed her newborn, we considered the breast “full” independent of the actual milk level in the lobes.

**Figure 3:**
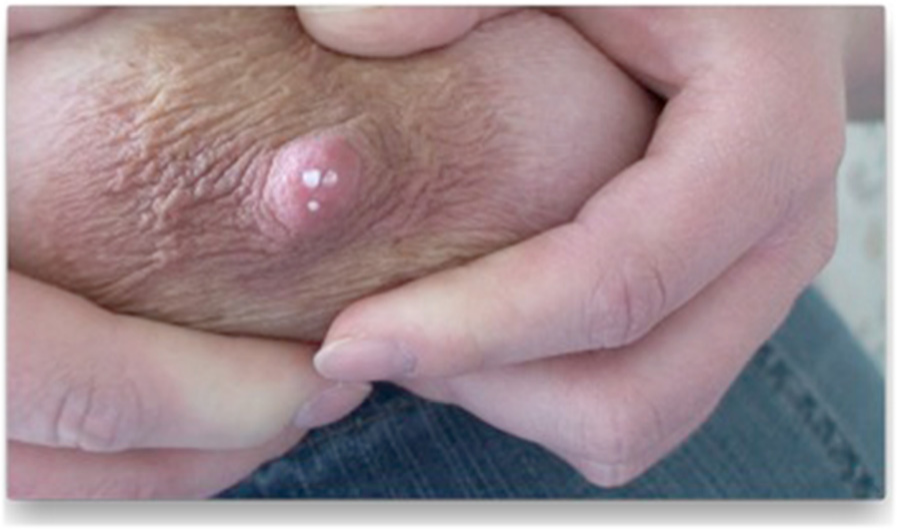
Manual milk expression was recorded with a HDR-SR 5E Handycam (Sony, Minato, Tokyo, Japan) to facilitate the identification of ductal orifices.

For the clinical identification of the milk duct orifices, direct observation during manual milk expression was performed. To achieve an exact discrimination of the orifices before pooling, two observers (JJ and a lactation consultant) examined the respective nipple. Video recording (HDR-SR 5E Handycam, Sony, Minato, Tokyo, Japan) was used to facilitate the evaluation. Mothers were not persuaded to have their breasts videotaped or assessed by the examiner JJ. Women who refused this process used self-examination for evaluation. Therefore, three options were used for data collection:

(i) Self-examination.

(ii) Personal examination by the investigator JJ and a lactation consultant during milk expression.

(iii) Video recording (HDR-SR 5E Handycam, Sony, Minato, Tokyo, Japan) by the investigator JJ during milk expression and subsequent analysis (Figure 3).

### Statistics

Descriptive and statistical analysis was performed using the software SPSS Statistics Release 21.0 (IBM Corporation, New York, U.S.A.). Continuous variables were reported by mean ± standard deviation and categorical variables by the number (n) and percentages. Different groups were compared using the Mann–Whitney U-test, in case of more than two groups, using the Kruskal-Wallis test. Left-hand and right-hand sides were compared using the Wilcoxon test. All are two-sided tests. The Spearman correlation coefficient was used to evaluate the relations between duct number and quantitative variables in the mother and the infant. P values smaller than 0.05 were considered statistically significant, values greater than or equal to 0.05, but smaller than 0.1 were interpreted as a statistical trend.

## Results

### Mothers

By the inclusion criteria 98 women were enrolled in this study, of whom 193 breasts (95 women both breasts, two women right breasts only, and one, left breast only) were observed during manual milk expression. The participants’ mean age was 32.8 ± 4.6 years (range 20–44). 62 women gave birth for the first time (age 32.2 ± 4.3 years); they were significantly younger (p = 0.028) than the other 36, multiparous women (age 33.9 ± 5.0 years). On the day of the interview, 62 women were employed and, due to pregnancy, were on leave for 1.0 ± 0.9 years on average.

### Infants

None of the enrolled mothers gave birth to multiples. Male infants (n = 55) were born after 39.8 ± 1.4 weeks of gestation; female infants (n = 43), after 39.6 ± 1.3 weeks of gestation. Birth weight, birth height, and age on the day of the interview are given in Table 1.

**Table 1:** Infants’ sex, birth weight, birth height and age at the day of the interview

	**Male (n = 55)**	**Female (n = 43)**	**p**
Weight (g)	3454.7 ± 490.0	3388.3 ± 502.6	n.s.
Height (cm)	51.9 ± 2.6	51.0 ± 2.3	0.079
Age (days)	63.7 ± 75.7	76.6 ± 89.0	n.s.

### Breastfeeding

The average time of every breastfeeding session across all infants was 23.1 ± 12.8 minutes. This time was registered as the duration in minutes of one breastfeeding session (left and right breast) up to when the infant did not actively request more milk. Male infants had significantly longer feeding sessions than female infants (Table 2). No differences associated with infants’ sex were found as to mother’s fluid intake, restless infant behavior during breastfeeding, and breast or nipple pain (Table 2).

**Table 2:** Different parameters of breastfeeding in relation to infants’ sex

	**Male infants (n = 55)**	**Female infants (n = 43)**	**p**
Breastfeeding time (min)	25.9 ± 14.1	19.6 ± 9.8	0.023
Breastfeeding sessions / 24 h	7.7 ± 2.5	8.3 ± 2.6	n.s.
Mother’s fluid intake (l / 24 h)	2.4 ± 0.6	2.4 ± 0.7	n.s.
Restless infant (%)	21.8%	16.3%	n.s.
Pain (%)	21.8%	32.6%	n.s.

### Ductal orifices

The mean number of ductal orifices determined in 95 women both whose nipples were observed was 7.6 ± 2.9 with a range of 2–16 orifices. On left nipples 3.8 ± 1.6 ductal orifices could be determined, right nipples exhibited 3.9 ± 1.6 (Table 3). There is no significant difference between the sides. The different methods for determining the ductal orifices showed no significant differences between the groups (p = 0.324).

**Table 3:** Distribution (n) of number of duct orifices in 193 nipples

**Orifices**	**n right**	**% right**	**n left**	**% left**
1	7	7.2	6	6.3
2	8	8.2	17	17.7
3	28	28.9	21	21.9
4	22	22.7	21	21.9
5	17	17.5	15	15.6
6	8	8.2	10	10.4
7	5	5.2	5	5.2
8	2	2.1	1	1.0
sum	97	100	96	100

Looking at the mean number of total ductal orifices in relation to infants’ sex (Table 4) reveals significantly more orifices on right nipples in women with male infants (p = 0.030).

**Table 4:** Mean number and standard deviations of ductal orifice number related to infants’ sex and left or right breast

	**Male infants**	**Female infants**	**p**
Left nipples	3.8 ± 1.7	3.8 ± 1.6	n.s.
Right nipples	4.2 ± 1.7	3.5 ± 1.4	0.030

Primipara and multipara as groups exhibited differences in age and number of ductal orifices (Table 5). The younger primipara had less orifices than the older multipara group (p = 0.022). No difference in ductal orifice number existed between mothers with painful nursing sessions and mothers without problems. The use of a breast pump had no effect either (Table 5).

**Table 5:** Mean number of total ductal orifices in primi- and multiparous women, women with and without pain during breastfeeding, and use of breast pump

	**Yes**	**No**	**p**
Primipara	7.1 ± 2.7	8.5 ± 3.0	0.022
Pain	7.1 ± 3.0	7.8 ± 2.9	N.s.
Use of breast pump	7.9 ± 2.9	7.5 ± 2.9	N.s.

**Figure 4:**
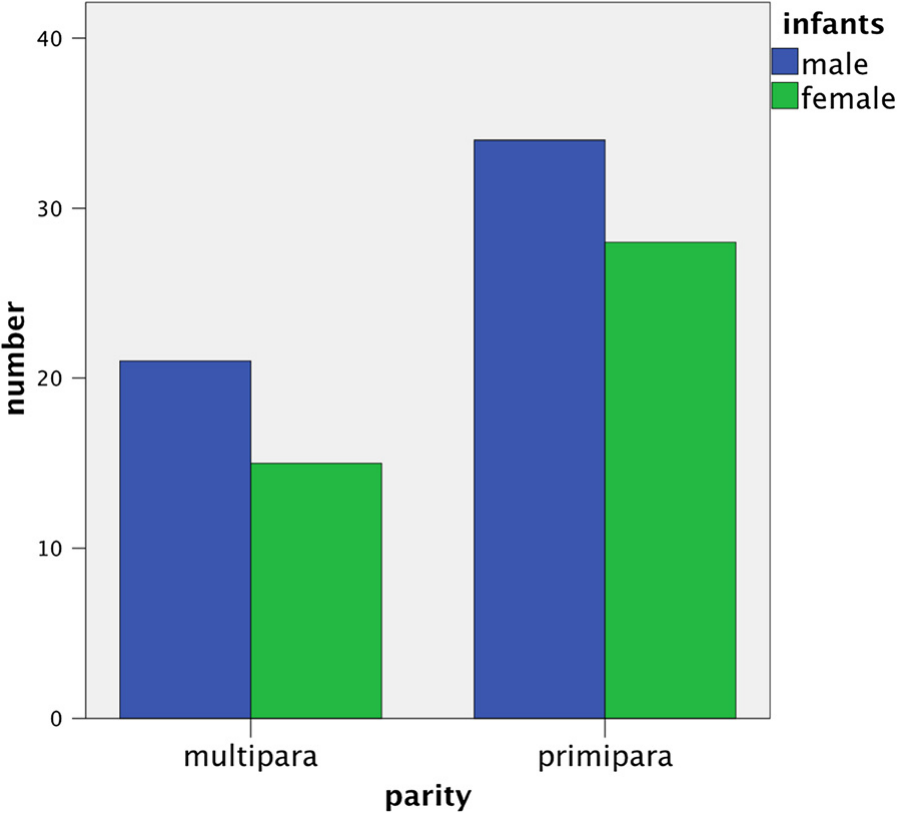
The distribution of male and female infant is comparable across both groups.

As to ductal orifices there is no correlation between male infants and multipara. The distribution of infants’ sex is comparable in both groups (Figure 4).

### Correlations

The evaluation of the number of ductal orifices as correlated with the assessed variables (Table 6) showed that a woman’s fluid intake was associated with more orifices in the right nipple (p = 0.028). Lower birth height of boys correlated with more ductal orifices in the left nipple (p = 0.081). Male’s feeding time was associated with more ductal orifices in right nipples too (p = 0.049). No other relations to ductal orifice number were found (Table 6).

**Table 6:** Spearman’s correlation coefficients and level of significance between orifices of each nipple and various obtained parameters

	**Orifices left**	**Orifices right**
Woman’s age	r = 0.153	r = 0.024
Weeks of gestation	r = -0.003	r = 0.037
Woman’s fluid intake	r = 0.076	r = 0.238; p = 0.028
Maternity leave	r = 0.044	r = -0.056
Intended breastfeeding period	r = 0.122	r = 0.089
Female infant’s weight	r = 0.185	r = 0.220
Male infant’s weight	r = -0.109	r = 0.012
Female infant’s height	r = 0.167	r = 0.080
Male infant’s height	r = -0.242; p = 0.081	r = -0.062
Female infant’s age	r = 0.031	r = 0.016
Male infant’s age	r = -0.010	r = 0.155
Female feeding time	r = 0.076	r = 0.065
Male feeding time	r = -0.200	r = -0.266; p = 0.049

## Discussion

In this study we examined, in vivo and taking a functional approach, the number of ductal orifices, coursing to and appearing on the surface of the nipple. We found that on average lactating women had 7.6 ductal orifices, with 3.9 orifices found per nipple. There was no difference between left and right breasts. Our study was conducted in a calm environment and with no exposure to stress. This was very important because it is known that emotional stress affects oxytocin release, which in turn reduces the effectiveness of the milk ejection response, resulting in less milk let down [10].

### Strengths and limitations of the study

Breastfeeding is an emotional interaction between mother and child. One strength of our study is that no invasive or interfering measurement method was used to assess duct orifices immediately before breastfeeding. Moreover, the used Marmet technique is widely known by lactation consultants and is easy to use by mothers. Love and Barsky [11] showed that one observer looking through a breast pump is able to discriminate up to 17 ductal orifices in one nipple. Video documentation and/or direct observation by two observers improves this measurement.

However, a manual technique may bias the results because it does not guarantee the full potential of milk ejection. Ultrasound imaging and time measurements between feedings could standardize milk levels and flow rate. Yet they do not address the emotional readiness for interaction of mother and child.

As in any interview, random errors can occur in their documentation. This concern was partially mitigated by handing out the documentation sheets to the mothers, who reviewed their answers immediately after the interview.

Studies on breast carcinoma have focused on hormone levels and ductal lobular units [11,12]. A literature review shows that in vitro studies with surgically obtained specimens identified more ducts and suggested more orifices than in vivo observations [13]. Analyzing histologic sections of the base of the papilla mammaria, Going and Moffat identified 27 ducts. Of these 27 ducts only 7 exhibited a patent lumen on the surface and, therefore, were classified as lactiferous ducts [13]. Love and Barsky [11] as well as Taneri et al. [7] pointed out that discrepancies of the number of identified intramammary ducts and surface orifices may be due to morphological similarities between these ducts and sebaceous and sweat ducts. This may result in mistaking sebaceous or sweat ducts for milk ducts. They also pointed out the possibility for ducts to anastomose [7,11]. Ramsay et al. [5] identified a mean number of 9 milk ducts (range 4–18) in each lactating breast using ultrasound imaging. They described the course of the ducts under the areola and inside the nipple as being diverse and complicated rather than systematically arranged, with anastomosis ducts coursing beneath and across one another. They, however, did not state how many of the imaged ducts ran to the nipple surface and exhibited an orifice [5]. There is a consensus in the literature to the effect that the number of milk ducts inside the lactating breast identified by ultrasound imaging or histologic sectioning is higher than the number of orifices on the nipple surfaces [7,13-16].

There is hardly any published in vivo study that addresses the number of milk duct orifices under functional aspects and analyzes their association with variables in the infant and the mother. The only comparable investigation available is by Love and Barsky [11], who found 5 orifices on average in every nipple. The small difference between their and our results may be explained by the different methods used. Love and Barsky used breast pumps instead of the Marmet technique. Due to the compressibility of milk ducts [17] manual methods involve a risk of blocking ducts, the consequence being that milk is not expressed from all ducts holding milk.

When comparing this to other, previously mentioned methods, this difference of results, however, is fairly small. A potential explanation for this may be the similarity of the approach, privileging a functional method of milk duct activation. We also found that the use of a breast pump in addition to breastfeeding did not influence the number of ductal orifices.

The question may be raised as to whether the number of ducts carrying milk during lactation affects breastfeeding parameters in the mother and the child and vice versa. In vivo studies about duct orifices are not available in the literature for comparison, but similarities could be found in studies that addressed the productivity of the breast [18,19]. In our study we used no methods to measure the productivity of the breast. We examined immediately before breastfeeding, when mothers felt they had enough milk to feed her babies. Therefore, causal relationships between the number of duct orifices and milk productivity could not be advanced, but this needs discussion.

In this study primipara were significantly younger than multipara. We found that multipara had more ductal orifices than primipara. Looking at the women’s age did not reveal a correlation with the number of ductal orifices. Kent et al. [19] did not find an association of a mother’s age or parity with her produced milk volume. However, our results support the assumption that parity, rather than age, affects the number of ductal orifices.

Yet another outcome of this study was that mothers with male infants exhibited more orifices than mothers with female infants, but only in right nipple. At a mean 4.2 ductal orifices in the right nipple, mothers of boys exhibited statistically significantly more orifices than mothers of girls, at a mean 3.5. Although the difference in number of orifices between left and right nipples has not been explained in the literature, in 2007 Kent [18] described that in 70% of reviewed cases the right breast was more productive than the left one. This fact did not correspond to left- or right-handedness of the mother, and mothers did not tend to offer the more productive breast to the infant either [18]. Mitoulas et al. [20,21], when considering infants’ 24-hour milk intake, found that right breasts produced significantly more milk than left breasts. At this point, the question may be raised as to whether the number of activated ductal orifices that bring milk to the surface of the nipple is associated with the milk volume the breast produces. We found that birth height and birth weight did neither activate more lactiferous ducts nor resulted in more ductal orifices expressing on the nipple surface. On the contrary male’s birth height is correlated negatively with duct orifice number in the left nipple.

A further explanation why male infants may induce more orifices is that male infants were fed significantly longer per meal than female infants. All infants in this study were breastfed 8.0 times on average in 24 hours. Breastfeeding sessions with boys, at an average duration of 25.9 minutes, lasted longer than those with girls, at 19.6 minutes. Kent et al. [19], who analyzed a study population of mothers and infants comparable to ours, found a frequency of 7.9 times in 24 hours. In their study they concluded that during breastfeeding sessions boys had a greater maximum milk intake than girls. This was associated with a higher milk production in general in mothers of boys compared to that in mothers of girls. They did not find a correlation between produced milk volume and frequency of breastfeeding in 24 hours [19].

It has to be discussed if the criteria “male” and “right breast” have the power to explain the higher number of ductal orifices. We found that lower birth height in boys correlated with more orifices in left nipples, which would oppose the assumption of a body side dependency. Looking at the male children, we found that primipara had less ductal orifices in their nipples than multipara, even though the number of male infants in primipara was higher than in multipara (Figure 4).

Women’s fluid intake in 24 hours correlated with more ductal orifices. This could be interpreted as conflicting with Dusdieker et al. who rejected a significant linear correlation between mothers’ fluid intake and produced milk volume [22].

The number of lactiferous ducts that expressed as milk pores on the surface of the nipple during our observation did not differ between mothers whose infants were restless and mothers whose infants were calm during breastfeeding sessions. Therefore, we conclude that their number does not affect the child’s behavior.

McClellan et al. [23] found that the majority of mothers feeling pain during nursing did not produce less milk than mothers without problems. We determined that breast sensitivity and pain was not linked to a lower number of milk ducts bringing milk into the infant’s mouth during breastfeeding. Pain did not result in longer or more frequent breastfeeding sessions either.

As previously mentioned, it is known that psychological aspects can affect successful breastfeeding and influence milk ejection [10,24]. Therefore, we included two psychological aspects in order to investigate if mothers’ personal breastfeeding intentions and attitudes affected the number of orifices. It is obvious that a longer maternity leave enables the mother to breastfeed her infant longer. Relying on that insight we expected that mothers planning to breastfeed their child for longer and being on a longer maternity leave would have more peace of mind, be more patient, feel less under pressure and stress when it comes to nursing, than mothers who deliberately step away from that breastfeeding intention. However, psychosocial aspects, e.g. duration of maternity leave or the intended total breastfeeding period, did not have any effect.

In summary, our results support the concept that the number of ductal orifices is a function of lactation. In this respect, Gooding et al. [25] as well as Going and Moffat [13] pointed out that regardless of the infant’s demand not all lobular structures and lactiferous ducts are activated during lactation because the overall milk-producing capacity of the breast exceeds the amount an infant needs.

## Conclusion

This is the first study to investigate in vivo the relation between the number of milk duct orifices in lactating breasts and different functional parameters in mothers and their infants.

The mean number of ductal orifices in 98 lactating woman was 7.6 ± 2.9, with 3.8 ± 1.6 orifices found in the left nipple and 3.9 ± 1.6 orifices in the right nipple. Multiparous women had more ductal orifices than primiparous women. Age of mother or infant had in general no effect on orifice number.

Although male infants were associated with more orifices in mothers’ right nipple and taller birth height in males was associated with less orifices on mothers’ left nipple, the effect of sex has to be interpreted with caution.

Longer breastfeeding time in males, higher fluid intake of mothers, and parity preferably speak for a functional and adaptive basis of nipple orifice number in lactating women as analyzed in vivo.

### Ethical approval

This investigation is part of a clinical trial registered under ClinicalTrial.gov (NTC00408746). The study was approved by the Ethics Committee of the Department of Medicine of the University of Münster, Germany. Written informed consent was obtained from all participants for data analysis and publication of the associated images. Permission to conduct the study was thereafter obtained from the following Departments of Gynecology:

– Klinik und Poliklinik für Frauenheilkunde und Geburtshilfe des Universitätsklinikums Münster.

– Klinik für Gynäkologie und Geburtshilfe des Knappschaftskrankenhauses Recklinghausen.

– Entbindungsstation der Klinik für Geburtshilfe der Paracelsus-Klinik Marl.

– Fachabteilung für Geburtshilfe des St. Vincenz-Krankenhauses Datteln.

The examination was performed in compliance with the Declaration of Helsinki, as amended, and with the regulations and guidelines of the International Conference for Harmonization of Good Clinical Practice (ICH-GCP).

## Competing interests

The authors declare that they have no competing interests.

## Authors’ contributions

TS and AH suggested the original idea for the paper, developed the study design and wrote the study protocol. JJ managed subject enrolment, ensured interviews and milk duct identification, collected the data and wrote parts of the manuscript. CS performed statistical analyses and contributed to the interpretation of the results. TS contributed to the statistical analysis and data handling and wrote the main part of the manuscript. DW wrote parts of the paper, researched the literature, contributed to the English translation, reviewed the paper’s content, including the final version of the manuscript. All authors read and approved the final manuscript.

## Pre-publication history

The pre-publication history for this paper can be accessed here:

http://www.biomedcentral.com/1471-2393/14/124/prepub
